# Endogenous attention modulates attentional and motor interference from distractors: evidence from behavioral and electrophysiological results

**DOI:** 10.3389/fpsyg.2015.00132

**Published:** 2015-02-20

**Authors:** Elisa Martín-Arévalo, Juan Lupiáñez, Fabiano Botta, Ana B. Chica

**Affiliations:** ^1^Department of Experimental Psychology, University of Granada, GranadaSpain; ^2^Mind, Brain, and Behavior Research Center, University of Granada, GranadaSpain; ^3^Lyon Neuroscience Research Centre, ImpAct team, INSERM U1028, CNRS UMR5292, LyonFrance

**Keywords:** distractors processing, interference, endogenous attention, event-related potentials (ERPs), Simon effect

## Abstract

Selective visual attention enhances the processing of relevant stimuli and filters out irrelevant stimuli and/or distractors. However, irrelevant information is sometimes processed, as demonstrated by the Simon effect (Simon and Rudell, 1967). We examined whether fully irrelevant distractors (task and target-irrelevant) produce interference (measured as the Simon effect), and whether endogenous orienting modulated this interference. Despite being fully irrelevant, distractors were attentionally coded (as reflected by the distractor-related N2pc component), and interfered with the processing of the target response (as reflected by the target-related lateralized readiness potential component). Distractors’ attentional capture depended on endogenous attention, and their interference with target responses was modulated by both endogenous attention and distractor location repetition. These results demonstrate both endogenous attentional and motor modulations over the Simon effect produced by fully irrelevant distractors.

## INTRODUCTION

Our environment contains more information than can be assimilated at a single glance. For this reason, a selective mechanism is crucial for an appropriate interaction with our environment. This mechanism would be essential to isolate important information, improve its perceptual and/or motor processing, and sometimes suppress irrelevant or distracting information (Desimone and Duncan, 1995). Researchers have traditionally considered attention as a mechanism for selection, biasing information processing in the brain. Attention leads to a selective perception of a small subset of the vast amount of information that continually inundates our senses (see e.g., Ruz and Lupiáñez, 2002), and/or biases action plans to the attended information (Allport, 1989).

However, despite attentional processes enhancing the representation of features that are task relevant, and/or suppressing task irrelevant features, the “competition” between attended and suppressed items (see e.g., Treue, 2001) is not an all-or-none phenomenon. In fact, the processing of irrelevant features of target stimuli and/or distractors can take place despite their irrelevance for the task at hand, as demonstrated in the stimulus–response (S–R) compatibility effect, better known as the *Simon effect* (Simon and Rudell, 1967; for a review). It consists of slower reaction times (RTs) to stimuli presented contralaterally to the predefined response location (i.e., incongruent condition) as compared to ipsilateral stimuli (i.e., congruent condition; see e.g., Lu and Proctor, 1995, for a review; see also Simon and Rudell, 1967). For example, in a discrimination task requiring responses to a laterally presented colored target, participants’ responses are faster when the spatial position of the stimulus (i.e., left or right) is ipsilateral to the position of the manual response key (i.e., either left or right) than when the stimulus is contralaterally presented. This effect is observed despite the fact that the spatial location of the target is completely irrelevant for the task at hand. Thus, the Simon effect reflects an interaction between the response-related spatial representation activated by the task-relevant dimension of the target (i.e., color) and the response-related spatial representation activated by the task-irrelevant dimension (e.g., location) of the target. It has been proposed that the effect is caused by an incongruence between two spatial S–R codes: one that is transiently generated from the spatial location of the target (which is task-irrelevant) and another one that, due to task-demands, would be activated by the non-spatial identity of the target (see e.g., Hommel, 1993; de Jong et al., 1994; Kornblum et al., 1999; Ivanoff et al., 2002).

Although some studies have demonstrated attentional influences on the Simon effect, no agreement has been reached about the role of spatial attention for the generation of the spatial code. This disagreement comes from the two major hypotheses explaining the effect: the attentional-shift hypothesis (see e.g., Umiltà and Nicoletti, 1990; Nicoletti and Umiltà, 1994), proposing a central role of spatial attention on the Simon effect, and the theory of event coding (TEC), proposing that spatial attention is not crucial for the generation of the spatial code in the Simon task, although it could modulate the Simon effect (see e.g., Hommel et al., 2001; Hommel, 2011).

Although many studies have so far explored the relationship between the Simon effect and the different attentional subsystems (see e.g., Hommel, 1993; Stoffer and Yakin, 1994; Pratt et al., 1997; Lupiáñez and Milliken, 1999; Ivanoff et al., 2002; Ivanoff and Klein, 2004; Lupiáñez and Funes, 2005; Van der Lubbe and Van der Helden, 2006; Abrahamse and Van der Lubbe, 2008; Luo et al., 2011), no agreement has been reached about the specific role of neither exogenous nor endogenous spatial attention in the context of the Simon effect. Most studies have explored exogenous attention (see e.g., Pratt et al., 1997; Lupiáñez and Solano, 1998; Lupiáñez and Milliken, 1999; Ivanoff et al., 2002; Ivanoff and Klein, 2004; Lupiáñez and Funes, 2005; Verleger et al., 2005; Luo et al., 2011), generally reporting an additive pattern (see e.g., Hommel, 1993; although see Van der Lubbe and Van der Helden, 2006). In the case of endogenous attention, while most studies have reported non-significant interactions between both factors (see e.g., Verfaellie et al., 1988; Proctor et al., 1992; Zimba and Brito, 1995; Wascher and Wolber, 2004), we are aware of a few studies that have observed a reduced Simon effect at endogenously attended locations as compared to unattended locations (see e.g., Abrahamse and Van der Lubbe, 2008; see also Umiltá and Liotti, 1987; Stoffer and Yakin, 1994; Van der Lubbe and Woestenburg, 1999). Thus, while the effect of exogenous attention is usually additive with the Simon effect (although see e.g., Van der Lubbe and Van der Helden, 2006), this result cannot be easily generalized to endogenous attention (see Klein and Ivanoff, 2011, for a review).

Interestingly, in all previous studies we are aware of, the Simon effect has always been investigated in relation to target locations, which although irrelevant for the task at hand, contained the relevant target for response. The novelty of the present study is to investigate whether fully irrelevant non-target stimuli, considered fully irrelevant distractors because they never shared any feature or location with the target, could modulate endogenous responses. Thereby, the current work presents a two-fold aim investigation: (1) to explore the role of *fully irrelevant distractors* (i.e., task and target-irrelevant) and (2) to investigate the inconsistent role of endogenous spatial attention on the Simon effect. Results of the present research will demonstrate whether irrelevant distractors produce Simon effect interference, and whether this effect is attentional or motor. If the effect were attentional, then it should interact with endogenous attention (and modulate attentional event-related components, as discussed below).

We assessed the influence of attentional capture by irrelevant distractors and endogenous orienting on discrimination responses to a grating line orientation task (see e.g., Chica et al., 2014; for a review of attentional studies). A target appeared either above or below the fixation point, and either alone or accompanied by an irrelevant distractor. A symbolic precue (valid, invalid, or neutral) preceded the appearance of the target display. In Experiment 1, we manipulated distractor location (always presented on the horizontal axis, i.e., left or right) in order to investigate the Simon effect. We explored if the Simon effect was modulated by distractor location repetition by presenting distractors before target presentation and at the moment of target presentation. In Experiment 2, our purpose was to link behavioral indexes of interference (Simon effect) with their electrophysiological correlates, to gain a better understanding of the neural mechanism/s underlying this effect, and its interaction with endogenous attention. In particular, the lateralized readiness potential (LRP) component represents a well-known index of selective motor activation (Coles, 1989). This component has been considered an important marker for the Simon effect because it implies covert response activation, mainly generated within M1 (see e.g., Leuthold and Jentzsch, 2002). LRP indicates if the correct hand is covertly activated in relation to target responses (see also Leuthold, 2011; for a review), reflecting direct visuo-motor activation evoked by the stimulus. Moreover, other components such as the N2pc, are related to a benefit for correctly allocating attentional resources, facilitating further perceptual processing of stimuli (see e.g., Luck et al., 1994; Hopf et al., 2000; Vogel and Luck, 2000; Hickey et al., 2009). The N2pc reflects attentional selection of objects (see e.g., Hilimire et al., 2010, 2011, for different indexes of target and distractor processing), representing in the present study a direct measure of distractor processing. The component is always related to lateralized stimuli presented on the horizontal axis, where targets were never presented (see e.g., Eimer, 1996, for a review). In the present experiment, targets could not elicit the N2pc, because they always appeared in the vertical axis (see e.g., Hilimire et al., 2010, 2011).

These electrophysiological indexes, beside other attentional ones related to perceptual (P100, N100), or decisional processes (P300), could shed light on the mechanisms underlying the interactions between the Simon effect and endogenous spatial attention.

## EXPERIMENT 1

### METHODS

#### Participants

A total of 30 healthy volunteers participated in this experiment (two left-handed, 25 women, mean age of 20.6 years, SD = 1.58). Data from one participant were excluded from the analyses due to a high error rate (over 50%). All participants in this and the following experiment were naïve students from the University of Granada, who participated in the experiment for course credits. They reported having normal or corrected to normal vision. The experiment was conducted in accordance with the ethical guidelines laid down by the Department of Experimental Psychology, University of Granada, in accordance with the ethical standards of the 1964 Declaration of Helsinki.

#### Apparatus and stimuli display

**FIGURE 1:**
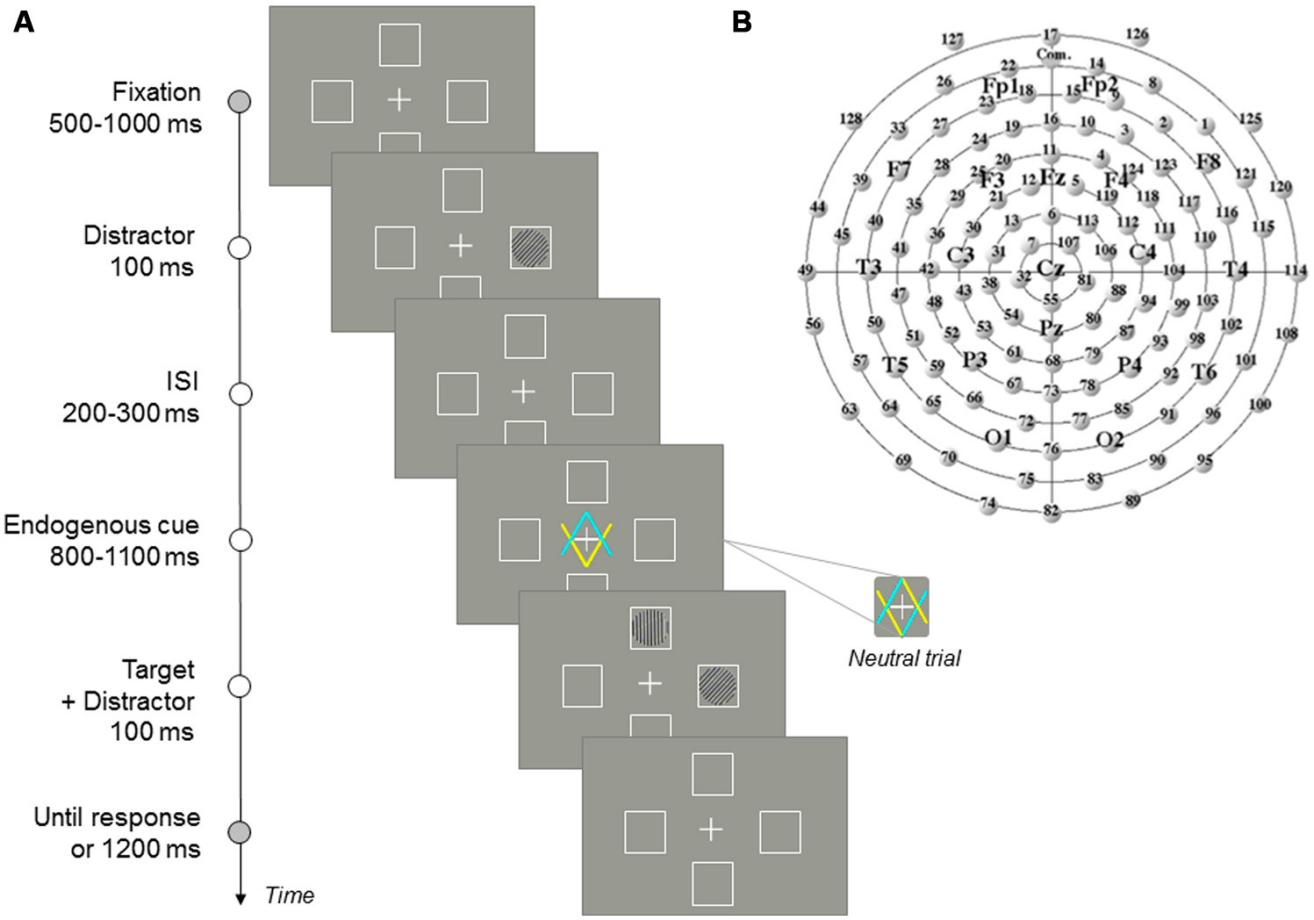
**(A)** Sequence of events in a given trial. **(B)** Sketch of the electrodes distribution around the scalp as viewed from above (the top of the figure represents the frontal area). Additional sites according to the 10–20 International system are shown for further reference.

The experiment was run on a computer with a 1GHz Pentium III processor, connected to a 15-inch color VGA monitor. E-prime 2 software (Schneider et al., 2002) controlled the presentation of stimuli and the acquisition of data throughout the experiments. All stimuli were white lines drawings presented against a gray background. Four placeholder boxes were presented around the fixation point (up, down, left, and right). Each box was 15 mm in width by 15 mm in height (subtending 1.5 and 1.5^∘^ of visual angle at a viewing distance of 57 cm, at which 1 cm corresponds to 1^∘^ of visual angle). Each box was positioned 3^∘^ away from central fixation along the horizontal and the vertical plane, as measured from the center of the placeholder to the center of the screen. The central fixation cross subtended 0.8^∘^ × 0.8^∘^. Distractors consisted of gratings subtending 1^∘^ in diameter, with left or right 45^∘^ titled stripes of 0.05^∘^, which appeared in the middle of one of the two placeholders boxes of the horizontal axis. The central cue was created by combining the central fixation cross with two overlapping arrowheads (1.7^∘^ × 1.7^∘^). One of the arrows pointed to the box on the upper side, while the other arrow pointed to the box on the lower side. Half of the participants were instructed to orient attention to the location indicated by the blue arrow, and the other half to the location indicated by the yellow arrow. For neutral trials, either the left or the right half of each arrow became yellow, and the other half blue. Corresponding left and right halves of the two arrows never had the same color (see **Figure 1**). Therefore, in neutral trials, central cues did not indicate any specific side of space (up/down). The target was a grating, subtending 1^∘^ in diameter, with vertical or horizontal stripes of 0.05^∘^, which appeared in the middle of one of the two placeholders of the vertical axis.

#### Procedure

The stimuli and sequence of events in each trial are illustrated in **Figure 1**. Each trial began with the presentation of a fixation display, containing the central fixation cross and the four placeholders, for a duration varying randomly between 500 and 1000 ms. Participants were required to keep gaze on the central fixation cross throughout the experiment. The distractor was presented in one of the two possible locations of the horizontal axis (left or right) for 100 ms. After distractor offset, another fixation display (with random duration between 200 and 300 ms) was presented. The central cue was then displayed for a random variable duration of 800–1100 ms. Half of the participants were instructed to pay attention to the box indicated by the yellow arrow, and the other half to the box indicated by the blue arrow. On directional trials (valid, in which targets are presented at the location indicated by the cue; invalid, in which targets are presented at the opposite location to that indicated by the cue; and directional catch trials, in which no target was presented and no response was required) the predictivity of this central cue (yellow or blue color) was 64.5%. Participants were informed about this predictivity and instructed to pay attention to the pointed location. On non-directional trials (neutral and neutral catch trials) participants were told that the central cues did not indicate any specific side of space (upper/lower), so that, in this case, they did not have to orient their attention to any specific marker. The target was then presented in one of the two possible locations of the vertical axis (upper or lower box). Simultaneously to target presentation, a distractor was again presented in one of the two possible locations of the horizontal axis. Responses were only required to the target, and therefore participants were instructed to ignore distractors. Participants were instructed to press the “z” or “m” keys on the keyboard to discriminate the orientation of the target stripes as quickly and accurate as possible. The target- response-key mapping (i.e., letter-key assignment) was counterbalanced across participants. After 100 ms, the target-distractor screen disappeared, and the fixation display was presented for 1200 ms or until a response was detected. The inter-trial interval, in which the screen remained empty, was 1500 ms in duration. An auditory feedback was presented for wrong, missing, or premature responses (shorter than 200 ms).

#### Design

The experiment consisted of a three-factor design. All variables were manipulated within participants. Validity (manipulated by the central endogenous cue) had three levels: valid, invalid, and neutral trials. Laterality (reflecting Simon congruency by presenting the second distractor at the time of target onset) had three levels: ipsilateral (i.e., distractor presented ipsilaterally to the target response -left/right manual key press), contralateral (i.e., distractor presented contralaterally to the target response), or distractor absent. Distractor location repetition had two levels: repeated location vs. non-repeated location (relative to the location of the first distractor). The experiments consisted of a total of 775 trials (500 trials were valid, 100 invalid, 100 neutral, and 75 catch trials). A practice block of 10 trials preceded the experimental trials. Practice trials were not further analyzed.

### RESULTS AND DISCUSSION

Participants missed the target (i.e., no response was detected) on 1.59% of the trials. Incorrect responses (5.95%) were excluded from the RT analysis. False alarms (i.e., responses to catch trials; 1.81%) and anticipations (responses faster than 200 ms; 1.61%) were also excluded from the RT analysis.

**Table 1** shows the mean RTs and the mean percentage of errors for each experimental condition. Mean correct RT data were submitted to a mixed analysis of variance (ANOVA) with the following factors: 3 (Validity: invalid, neutral, and valid) and 3 (Laterality: distractor ipsilateral to the target response, contralateral, or distractor absent). The analysis revealed a significant main effect of Validity, *F*(2,56) = 27.89, MSE = 180067, *p* < 0.0001, $\eta_{p}^{2}$ = 0.49, with faster RTs for valid (559 ms) than neutral trials (587 ms; *t*-test, *p* < 0.0001), and faster RTs for neutral than invalid trials (623 ms; *t*-test, *p* < 0.0001). The difference between valid and invalid trials was also significant (*t*-test, *p* < 0.0001). The Laterality effect was also significant, *F*(2,56) = 5.72, MSE = 28395, *p* = 0.0054, $\eta_{p}^{2}$ = 0.17; *t*-tests revealed significantly faster responses for ipsilateral (588 ms) than contralateral distractors (596 ms; *p* = 0.0180), as well as faster responses for distractor absent conditions (584 ms) than contralateral distractors (*p* = 0.0014). The difference between ipsilateral distractor and distractor absent conditions did not reach significance (*t*-test, *p* = 0.1205). Finally, the interaction between Validity and Laterality was not significant, *F* < 1^1^.

**Table 1:** Mean RT (in ms) for each condition.

	Laterality				Distractor location repetition	
	Validity	Ipsilateral	Contralateral	Absent	Repeated	Non-repeated
Experiment 1	Invalid	**620** [6.0%]	**625** [7.4%]	**623** [6.8%]	**14.85** [1.7]	**-1.14** [**-**0.2]
	Neutral	**587** [5.5%]	**595** [5.3%]	**578** [4.5%]	**21.64** [0.2]	**-6.01** [**-**0.5]
	Valid	**558** [6.5%]	**567** [5.1%]	**551** [6.2%]	**5.55** [**-**1.1]	**11.34** [**-**1.8]
Experiment 2-Behavioral	Neutral	**569** [6.5%]	** 578** [5.2%]		**12.69** [0.5]	**4.19** [0.5]
	Valid	**526** [4.7%]	**535** [4.0%]		**6.54** [**-**2.3]	**11.59** [**-**2.6]
Experiment 2-EEG	Neutral	**480** [5.9%]	**483** [6.1%]		**6.38** [-0.2]	**-0.93** [0.5]
	Valid	**440** [5.0%]	**444** [5.4%]		**-1.17** [0.5]	**8.75** [0.2]

The mean percentage of errors is presented in squared brackets. The Simon effect is presented on the right panel, as a function of distractor location repetition.

A similar analysis of the percentage of errors showed a significant main effect of Validity, F(2,56) = 3.36, MSE = 0.0017, *p* = 0.0417, $\eta_{p}^{2}$ = 0.10, showing a larger percentage of errors for invalid trials (6.77%) than neutral and valid trials (5.11 and 5.94%, respectively). Neither the main effect of Laterality nor its interaction with Validity were significant, all *p*s > 0.27.

**FIGURE 2:**
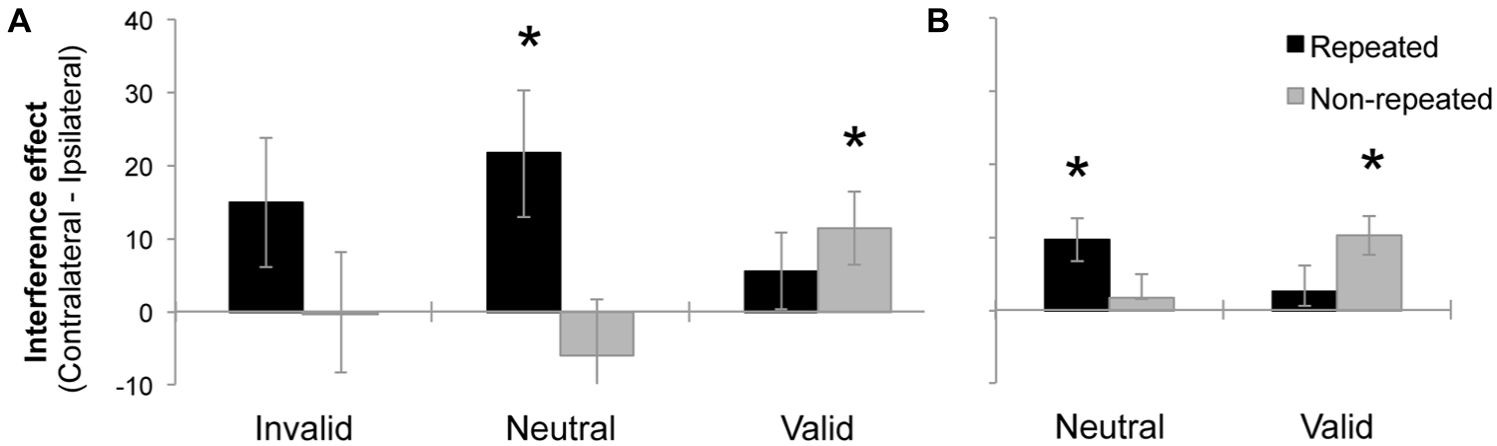
**Mean interference effect (mean RT for Contralateral minus Ipsilateral distractor conditions) as a function of Validity and Distractor location repetition, in the Experiment 1 **(A)** and Experiment 2 (B).** Error bars represent the standard error of the mean (SEM). Asterisks represent effects significantly differing from zero (*p* < 0.05).

After having demonstrated the presence of a significant Simon effect elicited by fully irrelevant distractors, we conducted a further ANOVA to investigate whether the Laterality effect was modulated by distractor location repetition and endogenous attention. A repeated-measures ANOVA was performed, with the following factors: 3 (Validity: invalid, neutral, and valid), and 2 (Distractor location repetition: repeated location vs. non-repeated location). The dependent variable in this analysis was an index of Simon interference (i.e., mean RT for contralateral minus ipsilateral distractors). The Validity effect was not significant, *F* < 1. The effect of distractor location was marginally significant, *F*(2,28) = 3.89, MSE = 1682, *p* = 0.0583, $\eta_{p}^{2}$ = 0.12, showing larger interference at the repeated location (14 ms) as compared to the non-repeated location (2 ms). Importantly, the interaction between validity and distractor location repetition was marginally significant, F(2,56) = 3.13, MSE = 1318, *p* = 0.0510, $\eta_{p}^{2}$ = 0.10. As it can be observed in the **Figure 2A**, we observed a significant interference effect for neutral trials when the distractor location repeated (*t*-test against zero, *p* = 0.0189). The same tendency was observed for invalid trials, although the effect did not reach significance (*t*-test against zero, *p* = 0.1058). For valid trials, the effect reversed, and interference was only observed when distractor location did not repeat (*t*-test against zero, *p* = 0.0286). None of the main effects or interactions were significant when the percentage of errors was analyzed, all *p*s > 0.15.

The results of the present experiment indicate that participants properly followed task-demands, endogenously attending to valid locations. Moreover, despite being fully irrelevant, distractors were also processed, as reflected by the Simon effect. The main result of this experiment was that interference depended on whether endogenous attention was focused. When attention was focused on valid trials, non-repeated location distractors produced the largest interference. However, when spatial attention was unfocused and/or more distributed (in neutral trials), repeated location distractors led to a larger interference than non-repeated location distractors. The implications of these data are discussed in the General discussion.

Due to the novelty of these findings, Experiment 2 had a two-fold aim: (1) to replicate the pattern of results observed in Experiment 1, and (2) to make use of electrophysiological measures to reach a better understanding of the neural mechanism/s underlying the interaction between endogenous attention and distractor processing.

## EXPERIMENT 2

This experiment replicated the design of Experiment 1 but eliminating the invalid attentional condition (which yield no significant modulation of the interference effect) and the distractor absent condition (which yield similar results to ipsilaterally presented distractors). We recorded high-density electroencephalogram to better understand the neural basis of interference generated by distractors, and its modulation by distractor location repetition and endogenous attention.

### METHODS

#### Participants

A total of 56 healthy volunteers participated in this experiment. Twenty-eight participants (all right-handed, all women, mean age of 22.1 years, SD = 2.67) took part in the behavioral task, while the remaining 28 participants (all right-handed, 20 women, mean age of 22 years, SD = 2.99) participated in the electroencephalographic (EEG) task. Data from five participants from the behavioral task were excluded from the analyses due to a high error rate (over 50%) or a technical error that prevented response recording. Data from one participant from the EEG task were also excluded from the analyses due to a high error rate (over 60%).

#### Procedure and design

The procedure and design were identical to Experiment 1, except for the following: Validity had two levels: neutral and valid trials. Laterality had also two levels: distractor ipsilateral vs. contralateral to the response hand. The central cue had a fixed duration of 500 ms, and a second ISI with a variable duration ranging randomly between 300 and 400 was presented. This ISI was introduced to get a better baseline for the target/distractor-locked ERP analysis. Finally, both tasks (i.e., behavioral and EEG) were identical, differing only in the number of total trials used; the former was composed of a total of 384 trials (192 trials per condition; valid vs. neutral) while in the latter, trials number was increased to a total of 640 trials (320 trials for each neutral and valid condition) to increase the signal to noise ratio for the EEG recording.

#### EEG task: recording and analysis

The electroencephalogram (EEG) was recorded using a 128-channel Geodesic Sensor Net of Ag/AgCl electrodes (Tucker et al., 1994). The head-coverage included sensors lateral to and below both eyes, to monitor horizontal and vertical eye movements. Impedances for each channel were measured and kept below 50 KΩ before testing. All electrodes were referenced to the Cz electrode during recording and were averaged re-referenced oﬄine. The EEG was amplified with a band pass of 0.1–100 Hz (elliptic filter), and digitized at a sampling rate of 250 Hz. EEG was filtered oﬄine by using a 30 Hz low-pass filter. Epochs were segmented from 200 ms before target/distractor appearance to 600 ms after its presentation. A 200 ms segment previous to the target/distractor presentation was used to calculate the baseline. All trials containing eye movements were corrected using *Ocular Artifact Removal* (OAR; Gratton et al., 1983). We rejected trials with blinks, artifacts, as well as trials with anticipatory responses. Data from three participants were also excluded from the analyses due to a reduced number of trials per condition after rejecting eye movements, blinks, and artifacts. For the remaining participants, an average of 11.75% of trials were excluded. A minimum of 25 trials per condition was required to ensure a sufficient signal-to-noise ratio.

### RESULTS

#### Behavioral results

Participants missed the target on 0.79% of the trials in the behavioral task, and 1.45% for the trials in the EEG task, which were no further analyzed. Incorrect responses (5.11 and 5.91% for the behavioral and EEG tasks, respectively), as well as responses faster than 200 ms (0.01 and 0.85% for the behavioral and EEG task, respectively), were also excluded from the RT analysis.

**Table 1** shows the mean RTs and mean percentage of errors for each experimental condition. Mean correct RT data were submitted to an ANOVA with the following factors: 2 (Task: behavioral and EEG) × 2 (Validity: neutral and valid) × 2 (Laterality: distractor ipsilateral vs. contralateral to the response hand). The analysis revealed a significant main effect of Task, *F*(1,48) = 18.60, MSE = 21659, *p* < 0.0001, $\eta_{p}^{2}$ = 0.27, showing faster RTs in the EEG task (462 ms) than in the behavioral task (552 ms), most probably due to the larger number of trials in the EEG task. In fact, further analyses (splitting the number of trials into two blocks of 320 trials 586 each) demonstrated faster RTs in the second half of the experiment as compared to the first half (*p* < 0.0001). However, Task did not interact with any other factor, all *p*s > 0.1146. There was a significant main effect of Validity, *F*(1,48) = 84.04, MSE = 993, *p* < 0.0001, $\eta_{p}^{2}$ = 0.63, with faster RTs for valid (486 ms) than neutral conditions (527 ms). The Laterality effect was also significant, *F*(1,48) = 14.26, MSE = 130, *p* = 0.0004, $\eta_{p}^{2}$ = 0.22, showing slower RTs for contralateral (510 ms) than ipsilateral distractors (504 ms). The interaction between Validity and Laterality was not significant, *F* < 1.

The analysis of the mean percentage of errors showed a significant main effect of Validity, *F*(1,48) = 12.82, MSE = 0.0005, *p* = 0.0007, $\eta_{p}^{2}$ = 0.21, with a larger percentage of errors for neutral (5.94%) than valid trials (4.79%). The interaction between Laterality and Task reached significance, *F*(1,48) = 7.48, MSE = 0.0002, *p* = 0.0086, $\eta_{p}^{2}$ = 0.13, showing a larger percentage of errors for the EEG task (5.77%) than for the behavioral task (4.61%) for contralateral distractors, while no differences were observed for ipsilateral distractors (5.47 and 5.61%, respectively). None of the other effects were significant, all *p*s > 0.1550.

As we did in Experiment 1, we carried out a second ANOVA with the Simon effect (i.e., contralateral minus ipsilateral distractors) as dependent variable. The following factors were manipulated within participants: 2 (Task: behavioral and EEG), 2 (Validity: neutral and valid), and 2 (Distractor location repetition: repeated location vs. non-repeated location). The analysis demonstrated that none of the main effects were significant, all *p*s > 0.0994. The interaction between Validity and Distractor location repetition was significant, *F*(1,48) = 9.54, MSE = 308, *p* = 0.0033, $\eta_{p}^{2}$ = 0.16, replicating the pattern of results observed in Experiment 1. As it can be observed in **Figure 2B**, we observed a significant interference effect for neutral trials when the distractor location repeated (*t*-test against zero, *p* = 0.0025). For valid trials, the effect reversed, showing interference only when distractor location did not repeat (*t*-test against zero, *p* = 0.0004). None of the other interactions were significant, all *p*s > 0.6488.

For the analysis of the mean percentage of errors, the main effect of Task was significant, *F*(1,48) = 7.34, MSE = 0.0011, *p* = 0.0092, $\eta_{p}^{2}$ = 0.13, showing an interference effect only in the EEG task. Moreover, the Validity effect was also significant, *F*(1,48) = 5.43, MSE = 0.0016, *p* = 0.0240, $\eta_{p}^{2}$ = 0.10, mirroring the interference effect observed in RTs. The interaction between Validity and Task was also significant, *F*(1,48) = 8.03, MSE = 0.0016, *p* = 0.0067, $\eta_{p}^{2}$ = 0.14, showing a larger interference for valid trials in the EEG task as compared to the behavioral task (error rate 0.4 and -2.5, respectively); no differences were observed in neutral trials between both experiments (error rate 0.1 and 0.4, respectively). None of the other main effects or interactions were significant, all *p*s > 0.5201.

#### ERP results

We analyzed event-related potentials (ERPs) locked to the appearance of the target/distractor. Five main ERP components were identified based on a visual inspection of the target/distractor-related grand average waveforms and topographic maps, according to previous literature (see e.g., Coles, 1989; Luck, 1995; Eimer, 2000; Hilimire et al., 2010, 2011). The first component was the P100, peaking at ∼140 ms, and observed in lateral occipito-parietal electrodes. This component was followed by a lateral occipital negativity (i.e., the N100), peaking at ∼210 ms in lateral occipito-parietal electrodes. Around ∼260–300 ms, the wave was more negative for contralateral than for ipsilateral occipito-parietal electrodes related to the distractor side. This negative difference (i.e., the N2pc component) was computed by subtracting the amplitude of the wave for electrodes contralateral minus ipsilateral to the distractor side. The N2pc is a lateralized component that appears over the visual cortex, contralateral to the attended location (either left or right); therefore, given our design, this component would exclusively reflect attraction of attention by the distractor, because only distractors were lateralized (see e.g., Eimer, 1996; Hilimire et al., 2011). We also observed a P300 component, peaking at ∼420 ms at central and occipital electrodes. Finally, we observed a lateralized larger negativity for contralateral than ipsilateral distractors at central electrodes (C3/C4; i.e., the LRP component). The LRP appeared 200–600 ms after target/distractor onset, according to previous literature (see e.g., Coles, 1989).

We calculated the mean amplitude of the P100 component (time window from 100 to 200 ms after target onset), N100 (time window from 160 to 260 ms), N2pc (time window from 250 to 350 ms), and P300 (time window from 370 to 470 ms), for each participant in a sample of representative electrodes covering the scalp (PO7/PO8, P3/P4, T5/T6, Pz/Cz, electrodes 12/5 representing Fz, F7/F8, Fp1/Fp2; see **Figure 1B**; see Chica et al., 2012, for similar analysis; see also Martín-Arévalo et al., 2014). In order to objectively determine the scalp location where each component was maximally elicited, we performed a one-way ANOVA for each component, with Electrode as a within-participants factor. Significant amplitudes for each component were further analyzed using *post hoc* tests (Bonferroni corrected *t*-tests for paired samples). For the P100 component, the largest mean amplitude of the component was observed at PO7/PO8 electrodes followed by P3/P4 electrodes (*M* = 1.31 μ and *M* = 0.89 μ, respectively; both amplitudes statistically comparable, *p* = 0.0921). The largest mean amplitude of the N100 component was observed at P3/P4 electrodes followed by PO7/PO8 electrodes (*M* = -1.78 μ and *M* = -1.43 μ; both amplitudes statistically comparable; *p* = 0.5406). Although the largest mean amplitude of the N2pc component was observed in the Fz electrode and Cz electrode (*M* = 2.02 μ and *M* = 1.76 μ), followed by P3/P4 electrodes and PO7/PO8 electrodes (*M* = 1.22 μ and *M* = 0.90 μ; both amplitude statistically comparable, *p* = 0.8128), we choose P3/P4 and PO7/PO8 electrodes for the analyses because, as it has been noted elsewhere (see e.g., Mangun and Hillyard, 1991; Luck, 1995), the N2pc is widely distributed over the posterior regions of the scalp (i.e., over visual cortical areas; see e.g., Eimer, 1994; Prime and Jolicoeur, 2009; but see e.g., Cespón et al., 2012, for a distintion between N2pc and N2cc, at central electrodes; see also Cespón et al., 2013). The largest mean amplitude of the P300 component was observed in the Pz electrode followed by P3/P4 electrodes (*M* = 5.67 μ and *M* = 4.27 μ; marginally different, *p* = 0.0662). Finally, we calculated the LRP by averaging the mean amplitude of the wave over the left and right C3 and C4 electrodes, and subtracting contralateral minus ipsilateral activity for each response hand depending on the distractor location (i.e., [(C4 – C3) _left-hand_ + (C3 – C4) _right-hand_]/2; see Coles, 1989, for a review about the method).

We subsequently analyzed the modulation of each component by calculating either its adaptive mean amplitude (20 ms before and after the higher peak; see Chica et al., 2012; Martín-Arévalo et al., 2014) at those electrodes and time windows where the components were maximally elicited based on the previous analyses (PO7/PO8 and P3/P4 electrodes for P100, PO7/PO8 and P3/P4 electrodes for N100, and Pz electrode for P300) or its mean amplitude in those components without a clearly defined peak (PO7/PO8 and P3/P4 electrodes for N2pc, and C3/C4 for LRP).

We carried out a mixed-design ANOVA on the mean amplitude of each component considering Validity (neutral and valid), Laterality (distractor ipsilateral vs. contralateral to the response hand), and Distractor location repetition (repeated location vs. non-repeated location) as within-participants factors. **Table 2** summarizes the results for each component.

**Table 2:** Results of the mean amplitude repeated-measures ANOVA with Validity (neutral and valid), Laterality (ipsilateral and contralateral), and Distractor location repetition (repeated location vs. non-repeated location) as factors.

	P100	N100	N2pc	P300	LRP	
Validity	n.s	n.s	n.s	*F*(1,23) = 13.84, MSE = 0.4901, *p* < 0.01, $\eta_{p}^{2}$ = 0.37	*F*(1,23) = 6.66, MSE = 0.1768, *p* = 0.0166, $\eta_{p}^{2}$ = 0.22	
Laterality	n.s	n.s	*F*(1,23) = 4.31, MSE = 0.1614, *p* = 0.0492, $\eta_{p}^{2}$ = 0.15	n.s	*F*(1,23) = 6.50, MSE = 0.6941, *p* = 0.0178, $\eta_{p}^{2}$ = 0.22	
Validity × Laterality	n.s	n.s	*F*(1,23) = 6.46, MSE = 0.1550, *p* = 0.0182, $\eta_{p}^{2}$ = 0.21	n.s	n.s	
Validity × Laterality × Distractor location repetition	n.s	n.s	n.s	n.s	*F*(1,23) = 4.37, MSE = 0.2125, *p* = 0.0477, $\eta_{p}^{2}$ = 0.22	
	All Fs < 1	All ps > 0.1863	All other ps > 0.1483	All other ps > 0.3043	All other ps > 0.3136	

Significant main effects and interactions are reported. n.s.: non-significant effect, p > 0.05.

### P100 AND N100

No main effect or interaction was significant either in the P100 or the N100 components^2^ (see **Table 2**).

### N2pc

**FIGURE 3:**
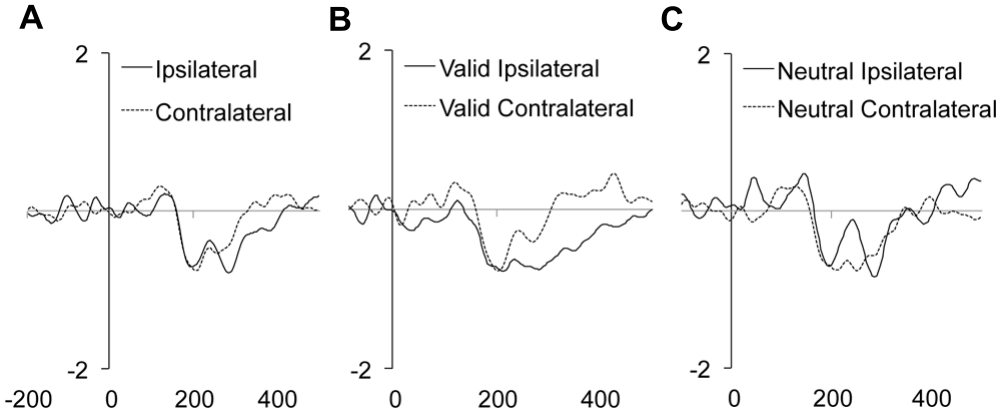
**Mean target/distractor-locked ERP waveforms for the N2pc component analysis for each condition of Laterality **(A)**, and Laterality × Validity (B,C)**.

Neither the main effect of Validity nor the main effect of Distractor location repetition reached significance. However, the main effect of Laterality was significant (see **Table 2**). As it can be observed in the **Figure 3A**, we observed a larger N2pc for ipsilateral as compared to contralateral distractor locations to the response hand. Laterality significantly interacted with the Validity effect, showing a significantly larger N2pc for ipsilateral as compared to contralateral distractor locations for valid trials (planned comparison, *p* = 0.0102; see **Figure 3B**), while the effect was non-significant for neutral trials (planned comparison, *p* = 0.7646; see **Figure 3C**)^3^. None of the other interactions reached significance. The implications of these data are discussed in the General discussion.

### P300

The main effect of Validity was significant (see **Table 2**), showing a larger P300 for targets appearing at neutral as compared to valid locations. None of the other main effects or interactions were significant, all *p*s > 0.3043.

### LATERALIZED READINESS POTENTIAL (LRP)

**FIGURE 4:**
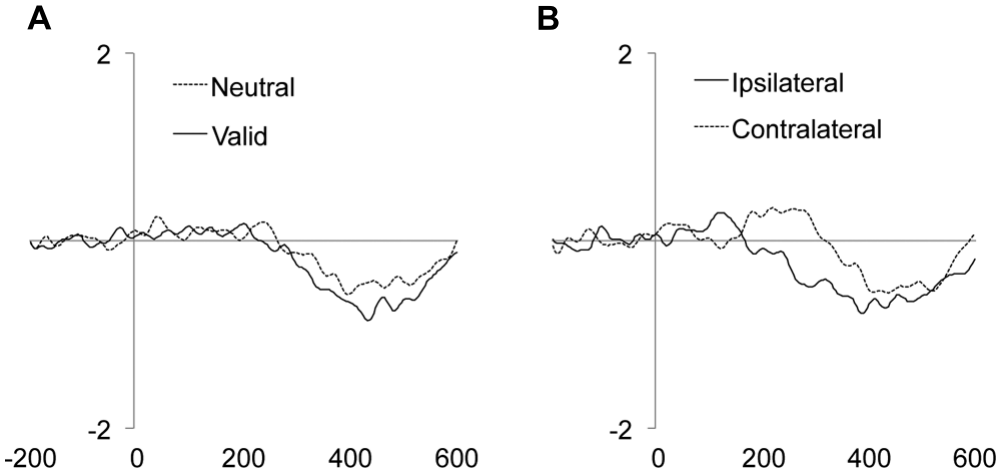
**Mean target/distractor-locked ERP waveforms for the LRP component analysis for each condition of Validity **(A)** and Laterality (B)**.

**FIGURE 5:**
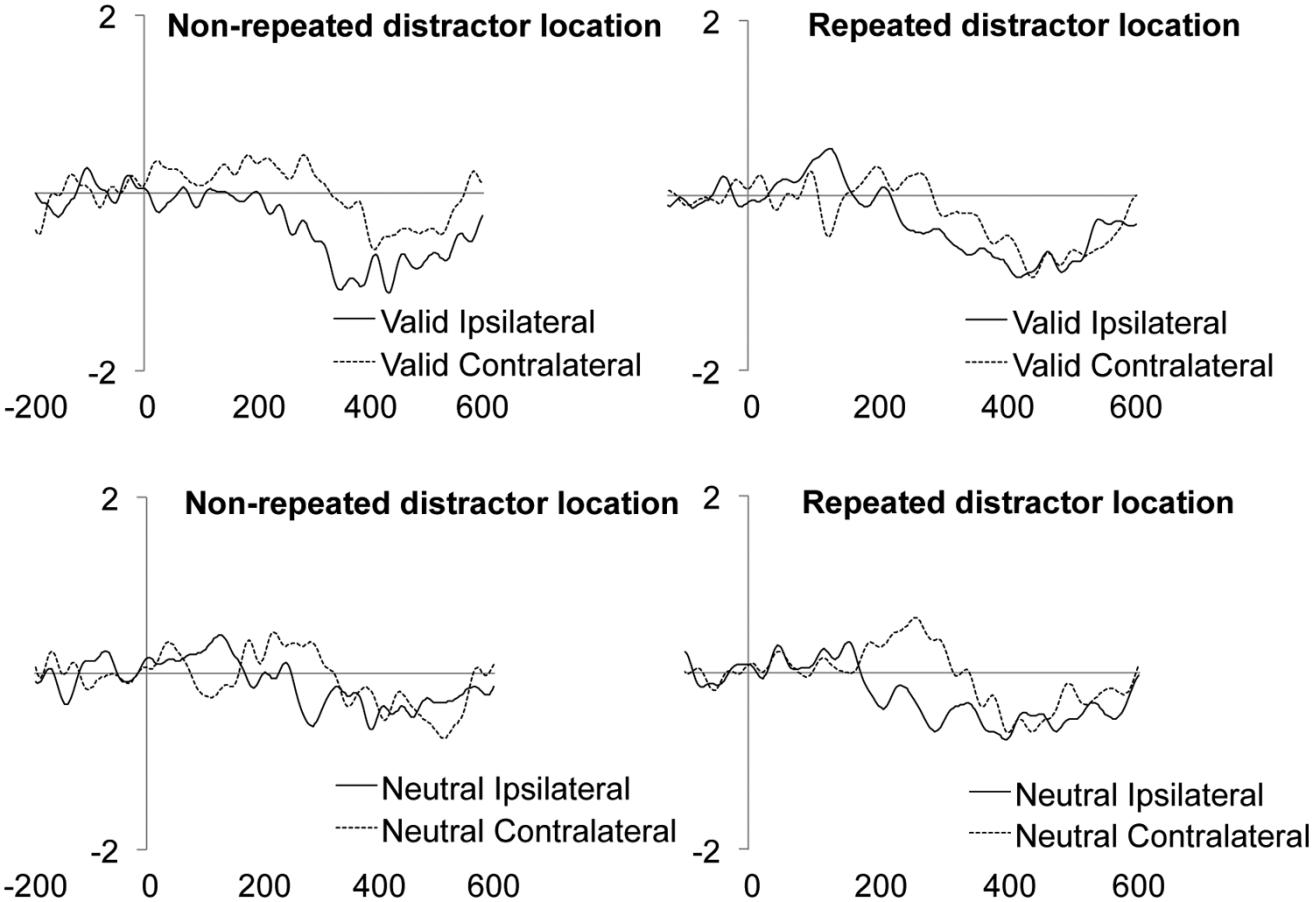
**Mean target/distractor-locked ERP waveforms for the LRP component analysis for each condition of Validity × Distractor location repetition × Laterality**.

The main effect of the Validity was significant, showing an enhanced amplitude of the LRP component for targets appearing at valid as compared to neutral locations (**Figure 4A** and **Table 2**). The main effect of Laterality was also significant, with larger LRP amplitude for targets accompanied by distractors ipsilateral to the response hand as compared to contralateral distractors (**Figure 4B** and **Table 2**). The interaction between Validity, Laterality, and Distractor location repetition also reached significance (see **Table 2**). As can be observed in **Figure 5**, for valid trials, larger differences between ipsilateral and contralateral distractors were observed when distractor location did not repeat (*t*-test, *p* = 0.0062) as compared to repeated distractor locations (*t*-test, *p* = 0.2271). For neutral trials, however, effects were not significant, although there was a trend for larger differences in the LRP amplitude for ipsilateral than for contralateral distractors when distractor location repeated (*t*-test, *p* = 0.0820) as compared to non-repeated locations (*t*-test, *p* = 0.4294). No other main effect or interaction was significant, all *p*s > 0.3136. The implications of these data are discussed in the General Discussion.

## GENERAL DISCUSSION

The present study aimed at investigating whether fully task irrelevant distractors (appearing at fully irrelevant spatial locations) produce interference (measured as the Simon effect), and whether endogenous attention modulates this effect. High-density EEG was used to explore whether these effects could be accounted for by the modulation of attentional (N2pc) or motor processes (LRP).

Previous studies have reported contradictory results for the interaction between the classic Simon effect and endogenous attention. While most of them have reported non-significant interactions between the two factors (see e.g., Verfaellie et al., 1988; Proctor et al., 1992; Zimba and Brito, 1995), a small number of studies have reported a reduction of the Simon effect at endogenously attended locations (see e.g., Abrahamse and Van der Lubbe, 2008; see also Umiltá and Liotti, 1987; Stoffer and Yakin, 1994). For example, Abrahamse and Van der Lubbe (2008) reported a reduction of the Simon effect at endogenously attended as compared to unattended locations. However, as it has been noted elsewhere (see e.g., Klein and Ivanoff, 2011), these experiments conform a set of design properties that could account for such findings: constant cue-target SOA, relatively short target durations, or targets presented at a larger than usual eccentricity (±8.3^∘^); wherein an explanation in terms of overt attention could not be completely ruled out. Additionally, the remaining studies that have shown a reduction in the Simon effect at the attended location have always used 100% valid cues and neutral cues (see e.g., Umiltá and Liotti, 1987; Stoffer and Yakin, 1994; Van der Lubbe and Woestenburg, 1999).

Our behavioral data are partially consistent with previous data showing no modulation of validity over the Simon effect, but also with previous data showing its reduction on valid trials: while we did not find a significant interaction between Validity and Laterality in any of the two experiments, thus conforming to most previous studies (see e.g., Verfaellie et al., 1988; Proctor et al., 1992; Zimba and Brito, 1995), we reported a reduction of the Simon effect at endogenously attended locations (see e.g., Abrahamse and Van der Lubbe, 2008; see also Umiltá and Liotti, 1987; Stoffer and Yakin, 1994, for similar results) when distractor location repeated as compared to non-repeated distractor locations. Thus, distractor location repetition seems to be a pivotal factor, at least in the present series of experiments, to disentangle the role of endogenous attention on the Simon effect. Our electrophysiological data are also contradictory with previous studies suggesting no N2pc modulations associated to the Simon effect (see e.g., Wascher and Wolber, 2004; Cespón et al., 2012, 2013), although coherent with some others demonstrating N2pc modulations (see e.g., Praamstra and Oostenveld, 2003; Hilimire et al., 2010, 2011).

Our results showed that N2pc was modulated by both, endogenous spatial attention and the Simon effect. This suggests a common attentional mechanism of spatial selection. However, as reviewed above, not all studies have consistently demonstrated an attentional modulation over the Simon effect, depending on the specific design used to manipulate attention and interference. We also observed no modulations in any of the components traditionally related to perceptual/attentional processing such as the P100 component and the N100 component (see e.g., Luck, 1995; Hopf et al., 2000). Taken all data together we can conclude that the existing evidence supports the theory of event coding (TEC) (see e.g., Hommel et al., 2001; Hommel, 2011), in which spatial attention is believed not to be crucial for the Simon effect.

Beyond procedure particularities and/or differences in results, the most important difference between all previous studies and the present one is that the above-mentioned studies have always investigated the Simon effect in relation to target features (i.e., target location, although task-irrelevant) while the present study was carried out in relation to completely task-irrelevant and target-irrelevant stimuli, i.e., distractors that never shared location or any other feature with the target. Therefore, comparisons between studies would not be entirely proper and should be done with caution. In the present experiments, the effect produced by distractors was exclusively due to sharing position with the target response location but never with the target location itself (which appeared in a different axis). Thus, while the previous studies produced interference by manipulating the irrelevant target location, we produced interference by manipulating the location of an irrelevant distractor that was presented together with the target; a novel approach to investigate both the Simon effect and its modulation by endogenous attention. Despite being fully irrelevant, distractors were spatially coded (as reflected by the N2pc component), and interfered with the processing of the target response (as reflected by the LRP component). Attentional capture by the distractor, as reflected in the N2pc component, and therefore its interference with target responses, was modulated by distractor location repetition as a function of endogenous attention.

These results lead to the conclusion, that, at least when the task requires selection of information, the Simon effect could be modulated by both endogenous attention and distractor location repetition. On the one hand, in relation to the distractor spatial processing itself, we observed that ipsilateral distractors led to higher distractor processing in terms of spatial selection. This finding fits with the contingent exogenous orienting hypothesis proposed by Folk et al. (1991, 1992). Ipsilateral distractors lead to a higher attentional capture because they share an attribute with the target, i.e., the response hand (see Folk et al., 1992, for a review). Interestingly, the amplitude of the N2pc component was enhanced for ipsilateral as compared to contralateral responses when attention was focused at the validly cued conditions, while no differences in laterality were observed in the neutrally cued conditions, when attention was not focused. Thus, when attention was endogenously focused (i.e., at valid trials) the Simon effect was observed and reflected as a larger attentional selection for ipsilateral than for contralateral distractor locations. Contrarily, when attention was not focused, as it is the case of neutral trials, the Simon effect was not significantly reflected in the N2pc, suggesting that the system could be equally ready for automatically responding to any position, no matter whether it was ipsilateral or contralateral to the target response hand.

Endogenous attention was also reflected in modulations of the LRP component. The larger LRP for valid than neutral conditions reveals a better motor selection for endogenously valid conditions. The LRP component also reflected the expected Simon effect, with larger LRP for ipsilateral than contralateral distractor positions, leading to a better motor selection when distractor location and response hand matched as compared to incongruent conditions, i.e., contralateral positions. Moreover, one of the most interesting findings observed in the present research was the interaction between Validity, Distractor location repetition, and Laterality; this interaction mirrored the behavioral effect reported in both experiments and demonstrated that interference produced by irrelevant distractors depended on endogenous attention. When attention was focused (at validly cued trials), the distractor affected motor stages of processing (LRP) but especially at non-repeated locations. This finding indicates that fully irrelevant distractors can produce both attentional and motor effects: distractors capture attention (as reflected in early stages of the N2pc component) and generate motor interference (as reflected in later stages of the LRP component) at non-repeated locations. When attention was not focused (at neutral cued trials), distractors did not produce effects at attentional stages of processing, but they tended to produce motor effects when distractor location repeated (although results did not reach statistical significance).

Although speculative, we propose that these novel results could be understood in terms of attentional resources (Lavie, 1995; see also Ruz and Lupiáñez, 2002, for a review) or priority allocation. As mentioned above, at valid conditions, where the system knows *a priori* the target position, it would have more free resources for processing new information (i.e., irrelevant distractors) at early stages, as indexed by the N2pc component. At late stages, indexed by the LRP component, the newest stimuli (non-repeated distractor locations) would lead to the largest interference. In neutral conditions, however, because the target position is unknown in advance, the system could be ready for processing any location, specially the two potential target locations. At the moment of target appearance, attentional resources could be dedicated to target processing, being prioritized and winning the competition with the distractor location. Thereby, at earlier stages, at which the N2pc is observed, there might not be free attentional resources for processing the distractor, thus explaining the absence of distractor laterality effects in the N2pc component on neutral trials. At later stages, where the LRP component is observed, repeated location distractors produce a larger interference, perhaps because the first distractor has not been properly filtered-out, and its effects are enhanced when distractor location repeats.

In summary, our results demonstrate that fully irrelevant distractors that do not share any feature with the target and/or task, capture our attention and modulate our responses, as reflected in different components related to distractor and target processing such as the N2pc component and the LRP component, respectively. When we endogenously attend to a spatial target location (preselecting it before the target appears), there is a larger *attentional selection* of the distractor (reflected in the N2pc); likewise, larger *motor interference* (reflected by the LRP) is also observed for the newest information (i.e., distractors presented at non-repeated locations). In contrast, when attention is unfocused (as it happens in neutral trials), the attentional resources would be more focalized to the target location at early processing stages, avoiding distractor processing. At later processing stages, *motor interference* is larger when distractor location repeats.

The present paper is, to the best of our knowledge, the first one showing how distractors that never share any feature with the target modulate the Simon effect at endogenously attended and non-attended locations. We consider this work as a first step to achieve a better understanding about the interactions between the attentional and motor systems in relation to surprising or irrelevant stimuli.

## Conflict of Interest Statement

The authors declare that the research was conducted in the absence of any commercial or financial relationships that could be construed as a potential conflict of interest.

## Footnotes

1 In a previous analysis, we introduced Distractor line orientation repetition (same vs. different) as another within-participants factor. The main effect of distractor line orientation repetition was not significant and did not interact with any other variable, all *p*s > 0.0971. We therefore decided to report the data collapsing across this variable.

2 Data from PO7/PO8 and P3/P4 were collapsed because both amplitudes were statistically comparable (*t*-tests, *p* = 0.0921 and *p* = 0.5406 for P100 and N100, respectively).

3 Data from PO7/PO8 and P3/P4 were collapsed because the mean amplitude analyses showed no significant differences between the two electrodes groups (*t*-test, *p* = 0.8128).
